# N-glycomic Profile in Combat Related Post-Traumatic Stress Disorder

**DOI:** 10.3390/biom9120834

**Published:** 2019-12-06

**Authors:** Lucija Tudor, Gordana Nedic Erjavec, Matea Nikolac Perkovic, Marcela Konjevod, Dubravka Svob Strac, Suzana Uzun, Oliver Kozumplik, Tanja Jovanovic, Gordan Lauc, Nela Pivac

**Affiliations:** 1 Laboratory for Molecular Neuropsychiatry, Division of Molecular Medicine, Rudjer Boskovic Institute, 10000 Zagreb, Croatia; lucija.tudor@irb.hr (L.T.); gnedic@irb.hr (G.N.E.); mnikolac@irb.hr (M.N.P.); marcela.konjevod@irb.hr (M.K.); dsvob@irb.hr (D.S.S.); 2 Department for Biological Psychiatry and Psychogeriatrics, University Hospital Vrapce, 10000 Zagreb, Croatia; suzana.uzun@gmail.com (S.U.); okozumplik@hotmail.com (O.K.); 3 School of Medicine, Josip Juraj Strossmayer University of Osijek, 31000 Osijek, Croatia; 4 Psychiatry and Behavioral Neurosciences, Wayne State University Detroit, MI 48202, USA; tjovano@emory.edu; 5 Genos Ltd., Glycobiology Laboratory, 10000 Zagreb, Croatia; glauc@genos.hr

**Keywords:** biomarkers, Caucasians, immunoglobulin G, male subjects, N-glycans, plasma, PTSD, veterans

## Abstract

Post-traumatic stress disorder (PTSD) develops in a portion of individuals exposed to extreme trauma. Glycosylation is a post-translational modification that affects protein functions and is altered in various pathophysiological states and aging. There are still no validated biomarkers of PTSD. The aim of this study was to evaluate the N-glycomic profile in 543 male Caucasian individuals (299 veterans with PTSD and 244 control subjects). The study included discovery (*N* = 233) and replication (*N* = 310) cohort. Hydrophilic interaction HPLC and ultra-performance liquid chromatography were used to separate and detect 39 plasma and 24 IgG N-glycan species, respectively. All results were corrected for the effects of age and multiple testing. Significant results included only significantly altered N-glycans in cases/controls in both cohorts, in the same direction. Results showed that six plasma N-glycans (four increased and two decreased) were altered in PTSD vs. controls in both cohorts, but IgG N-glycans were similar between groups. The severity of PTSD was not associated with different plasma N-glycans. This is the first study detecting alterations in plasma N-glycans in PTSD. These N-glycans are also associated with other neuropsychiatric disorders and inflammation, suggesting possible shared glycosylation mechanisms.

## 1. Introduction

Post-traumatic stress disorder (PTSD) is a trauma- and stressor-related disorder [1] that develops in a portion of individuals who show distress and functional impairment after experiencing or witnessing a traumatic event. PTSD symptom clusters include re-experiencing, avoidance, negative thoughts, negative mood, and hyperarousal [1]. The prevalence of PTSD differs according to differences in trauma exposure and trauma types worldwide and can be regionally specific. Combat and war-related traumas are associated with 11–20% prevalence in United States combat veterans [2], whereas in Croatian combat exposed veterans, the estimated prevalence of PTSD ranges from 14 to 40% [3]. PTSD is associated with different somatic comorbidities such as metabolic syndrome, increased inflammatory processes, pulmonary, cardiovascular and autoimmune diseases [4,5,6,7], and it is recognized as systemic disease [8]. Due to its complex etiology, the biological underpinning of PTSD is still not clear, validated and clinically useful biomarkers have not been identified, and various potential suggested biomarkers were not replicated [9].

N-glycans are complex carbohydrate chains linked by N-glycosidic bonds to various proteins or lipids. Glycosylation is a post-translational modification that affects diverse protein functions such as cell adhesion, signal transduction, receptor activation, molecular trafficking, and systemic clearance [10]. N-glycans affect different cellular processes and biological functions [11]. Altered glycosylation in plasma, serum, and cerebrospinal fluid (CSF) is found in cancer, autoimmune, infectious and chronic inflammatory diseases [11,12] and aging [12,13,14]. N-glycans attached to IgG, constituting 10–20% of plasma proteins, have been associated with the pathogenesis and progression of different diseases, and biological age [12]. Beside these somatic disorders, changes in plasma, serum and CSF glycosylation patterns were found in different neuropsychiatric diseases, such as schizophrenia [15], major depressive disorder (MDD) [16], attention-deficit hyperactivity disorder (ADHD) [17], dementia [14] and Alzheimer’s disease (AD) [18]. To date, only one study evaluated 9 N-glycan structures and accelerated aging in a small number of trauma exposed subjects [19]. An association between particular protein glycosylation in highly stressed groups, such as in professional soldiers was detected in earlier study [20]. Although the authors did not use highly specific and robust N-glycosylation profiling, they reported a significantly higher concentration of the highly-sialylated glycoprotein stressin whose concentration was positively correlated with stress intensity [20]. 

PTSD is a severe disorder that significantly impairs normal functioning and quality of life of affected persons and their families [9]. Therefore, better understanding of the biological basis of PTSD is extremely important. The exact biological factors associated with vulnerability or resilience to PTSD are still unknown, but vulnerability is presumably the result of the complex interplay between environmental, biological, and genetic factors. Clinically useful and validated biomarkers of PTSD, that will enable insight into the biological alterations in PTSD, are missing [9]. N-glycans are usually considered as mediators between environmental stimuli and genetic background that are highly responsible for crucial physiological processes and are strongly affected by age [14], sex [21], and ethnicity [22]. Therefore, the aim of this study was to compare the N-glycosylation profile between war veterans with PTSD and age-, sex-, and ethnicity- matched healthy control subjects. The goal of the study was to identify validated and clinically useful biomarkers of PTSD. The hypothesis of this study was that alterations in plasma/IgG N-glycome will be associated with PTSD and/or severity of PTSD symptoms. We also predicted, based on the preliminary results of the accelerated aging in PTSD [19] that veterans with PTSD will have higher values on the GlycoAge Test than control subjects, indicating faster aging in PTSD.

## 2. Methods

### 2.1. Participants

The study design included two cohorts: discovery and replication, which together comprise 543 age-matched unrelated male Caucasian individuals of Croatian origin: 299 with combat-related PTSD and 244 age-matched control subjects, not exposed to combat trauma. Discovery cohort was enrolled in the period between autumn of 2015 and spring of 2016, whereas the samples for the replication cohort were collected in the period between autumn of 2016 and the spring of 2017. Individuals had current and chronic PTSD, diagnosed using the Structured Clinical Interview (SCID) based on DSM-5 criteria [1]. At the time of sampling, individuals with PTSD did not receive psychiatric medication for at least 30 days. Control (*N* = 73) and subjects with PTSD (*N* = 160) in the discovery cohort (*N* = 233) were sampled from October 2015 to January 2016, whereas control (*N* = 171) and subjects with PTSD (*N* = 139) in the replication cohort (*N* = 310) were sampled from October 2016 to February 2017. Individuals with PTSD were 39–77 years old, with exposure to warzone trauma during the Homeland war in Croatia (1991–1995). The median age (25th; 75th percentile) of PTSD patients was 55 (50; 59) and 56 (52; 53) in the discovery and replication cohort, respectively, whereas the median age (25th; 75th percentile) of control subjects was 53 (48; 58) and 57 (49; 63) in the discovery and replication cohort, respectively. The Clinician Administered PTSD Scale (CAPS) [23] was used to assess severity of PTSD. According to the CAPS severity, individuals with PTSD were subdivided into those with moderate (range: 66–95 CAPS scores) and severe (range: 96–136 CAPS scores) PTSD symptoms. There were no individuals with mild PTSD symptoms (range: 46–65 CAPS scores). Median number of CAPS scores (25th; 75th percentile) was 86 (78; 88) in the discovery cohort and 85 (78; 86) in the replication cohort. The median BMI (25th; 75th percentile) of healthy control subjects was 27.4 (25.7; 30.0) and 29.0 (27.2; 31.2) in the discovery and replication cohort, respectively; whereas median BMI (25th; 75th percentile) of individuals with PTSD was 27.7 (25.8; 30.3) and 28.1 (25.7; 30.5) in the discovery and replication cohort, respectively. 

Exclusion criteria were chronic drug abuse, alcohol dependence or pathophysiological changes in the liver, MDD, schizophrenia, bipolar disorder, adult ADHD, AD, current or recent (previous 3 months) use of lipid lowering agents, antihypertensive, and antidiabetic medication. All participants were additionally evaluated according to the International Classification of Diseases (ICD-10) to exclude potential somatic disease such as fibrosis, sclerosis, cirrhosis and malignant liver disease (alcoholic liver cirrhosis (K70.3), alcoholic liver fibrosis and sclerosis (K70.2), and hepatocellular carcinoma (C22.0)) that might affect N-glycan levels.

Healthy control subjects were evaluated with the same diagnostic instruments and followed the same inclusion/exclusion criteria. The aims and procedures of the study were explained in detail to all participants. Written informed consent was obtained from all subjects/patients. The authors assert that all procedures contributing to this work comply with the ethical standards of the relevant national and institutional committees on human experimentation and with the Helsinki Declaration of 1975, as revised in 2008. All procedures involving human subjects/patients were approved by the Ethics Committee of the University Psychiatric Hospital Vrapce, Zagreb, Croatia.

### 2.2. Blood Sampling 

Blood samples were collected between 7:30 and 8:00 a.m., after overnight fasting, using BD Vacutainer™ glass blood collection tubes (Becton, Dickinson and Company, Franklin Lakes, NJ, USA) with acid citrate dextrose (ACD). Immediately after blood sampling, platelet poor plasma was separated by series of centrifugation and stored at −80 °C. Plasma samples, in both cohorts, were randomized and blinded prior to N-glycan determination to avoid bias.

## 3. N-glycan Determination in Plasma 

### 3.1. Glycan Release and Labelling in Plasma

Plasma N-glycans were determined as described previously [24]. Plasma samples (10 μL) were mixed with 20 μL of 2% (*w*/*v*) SDS (Invitrogen, Camarillo, CA, USA) for 10 min at 65 °C in order to achieve protein denaturation in the samples, followed by addition of 4% (*v*/*v*) Igepal CA630 (SigmaAldrich, St. Louis, MO, USA) to the mixture. N-glycans were released by digestion with 1.2 U PNGase F (Promega, San Luis Obispo, CA, USA) overnight, at 37 °C. Following extraction, glycans were fluorescently labeled with 2-aminobenzamide (2-AB) after 2 h incubation at 65 °C. To prepare the labeling solution, 2-AB (19.2 mg/mL; SigmaAldrich, St. Louis, MO, USA) and 2-picoline borane (44.8 mg/mL; SigmaAldrich) were dissolved in a mixture of dimethylsulfoxide (SigmaAldrich, St. Louis, MO, USA) and glacial acetic acid (Merck, Darmstadtd, Germany) (70:30 *v*/*v*).

### 3.2. Hydrophilic Interaction High Performance Liquid Chromatography (HILIC)

Released glycans were subjected to HILIC, Acquity UPLC instrument (Waters, Milford, MA, USA), on a Waters BEH Glycan chromatography column, 150 × 2.1 mm i.d., 1.7 μm BEH particles at 25 °C with 100 mM ammonium formate adjusted to pH 4.4 (solvent A) and acetonitrile (solvent B). Samples were kept at 10 °C before the injection. The separation was achieved with a linear gradient of solvent A (30–47%) at 0.56 mL/min flow rate, during 23 min long analysis. Runs were performed with fluorescence detector set with excitation and emission wavelengths of 250 and 428 nm, respectively. The system was calibrated using an external standard of hydrolyzed and 2-AB-labeled glucose oligomers from which the retention times for the individual glycans were converted to glucose units. The obtained chromatograms were all separated into 39 chromatographic areas (peaks) and automatic processing method, with a traditional integration algorithm, was used to process the data. The amounts of glycan present in each area were expressed as the percent of total integrated chromatogram and the peaks were assigned as described by Saldova and colleagues [25].

## 4. N-glycan Determination in IgG

### 4.1. Glycan Release and Labelling in IgG

Plasma samples (50 μL) were diluted 10x with the binding buffer and applied to the Protein G plate. The filtration of the samples was completed in approximately 5 min. The plate was washed five times with 5 column volumes (CV) of binding buffer to remove unbound proteins. IgG was released from the protein G monoliths using 5 CV of elution solvent (0.1 M formic acid, pH 2.5). Eluates were collected in a 96 deep-well plate and neutralized to pH 7.0 with 1 M ammonium bicarbonate to maintain the IgG stability. After each sample application, the monoliths were regenerated with the following buffers: 10 CV of 10× PBS, followed by 10 CV of 0.1 M formic acid and 10 CV of 1× PBS to re-equilibrate the monoliths. Each step of the chromatographic procedure was done under vacuum (cca. 60 mmHg pressure reduction while applying the samples, 500 mmHg during elution and washing steps). The purity of the isolated IgG was verified by SDS-PAGE with NuPAGE Novex 4–12% Bis-Tris gels in an Xcell SureLock Mini-Cell (Invitrogen) according to the manufacturer. Precision Plus Protein All Blue Standards (BioRad, Hercules, CA, USA) were used as the molecular weight markers. The gels were run at 180 V for 45 min, stained with GelCode Blue (Thermo Fisher scientific, Waltham, MA, USA) and visualized by a VersaDoc Imaging System (BioRad, Hercules, CA, USA).

### 4.2. Ultraperformance Liquid Chromatography (UPLC) Analysis of IgG Glycans 

Fluorescently labelled N-glycans were separated by UPLC on a Waters Acquity UPLC instrument consisting of a quaternary solvent manager, sample manager and a FLR fluorescence detector set with excitation and emission wavelengths of 330 and 420 nm, respectively. The instrument was under the control of Empower 2 software, build 2145 (Waters, Milford, MA, USA). Labeled N-glycans were separated on a Waters BEH Glycan chromatography column, 100 × 2.1 mm i.d., 1.7 μm BEH particles, with 100 mM ammonium formate (pH 4.4) and acetonitrile. The separation method was developed, which uses a linear gradient of 75–62% acetonitrile at a flow rate of 0.4 mL/min in a 20 min analytical run. Samples were maintained at 5 °C prior to injection, and the separation temperature was 60 °C. The system used hydrolyzed and 2-AB labeled glucose oligomers as external standard, from which the retention times for the individual glycans were converted to glucose units. Data processing was performed using an automatic method with a traditional integration algorithm after which each chromatogram was manually corrected to maintain the same intervals of integration for all the samples. The obtained chromatograms were separated into 24 peaks representing percentage of total integrated area as described previously [14].

## 5. Statistical Analysis

To remove experimental variation from measurements, normalization and batch correction were performed on UPLC glycan data. Normalization of raw measurements within each sample is required because intensities of UPLC signals can vary more than 100-fold due to experimental noise. Also, laboratory conditions can vary during the experiment, so correction of batch effects is necessary [26]. To make measurements across samples comparable, normalization by total area is performed where peak area of each glycan structure was divided by total area of corresponding chromatogram. Prior to batch correction, normalized glycan measurements were log transformed due to right-skewness of their distributions and multiplicative nature of batch effects. Batch correction was performed on log-transformed measurements using ComBat method [27], R package sva [28], where technical source of variation (which sample was analyzed on which plate) was modeled as batch covariate. To get measurements corrected for experimental noise, estimated batch effects were subtracted from log-transformed measurements.

The results are expressed as medians and 25th (Q1) and 75th (Q3) percentiles and were evaluated with R Statistics 3.5.1. Normality of the distribution was assessed with the Kolmogorov–Smirnov test. As most of the glycan peaks were not normally distributed, non-parametric analyses were used. The 39 plasma and 24 IgG N-glycans are shown as percentages of the total area under the curve. Besides individual N-glycans in plasma and IgG, GlycoAge index was calculated for each individual. GlycoAge index was determined as a logarithmic ratio of two N-glycan structures that showed the highest changes in concentration depending on age: agalactosylated core-a-1,6-fucosylated biantennary N-glycan (FA2), which concentration increases gradually with age and bigalactosylated core-a-1,6-fucosylated biantennary N-glycan (FA2G2), which concentration decreases with aging (log10(FA2/FA2G2)) [14]. Plasma and IgG samples from the PTSD and control group are shown separately for the discovery and the replication study. Multiple linear regression was used to determine the effect of age, BMI and smoking on levels of plasma and IgG N-glycan species. As the age is a main predictor of N-glycosylation changes in human plasma [29] and IgG [30], correction for the effect of age was performed by fitting the linear model of each glycan peak in dependence of age and using the obtained residuals for further statistical analysis [19]. Differences in the distribution of N-glycans and GlycoAge index values between veterans with PTSD and control subjects, and between PTSD severity subgroups were evaluated using Mann-Whitney tests with corrected N-glycan values. Correlations between N-glycans and severity of PTSD were analyzed with Spearman’s rank tests. False discovery rate (Benjamini Hochberg) method was used to correct *p* values due to the multiple testing. The corrected values at *p* < 0.05 were considered significant.

## 6. Results

Multiple linear regression analyses revealed that in our sample smoking and BMI were not significantly associated with majority of N-glycans in plasma (*p* > 0.05) or IgG (*p* > 0.05). However, as expected, there was a strong association of N-glycans with age, and therefore all glycan values were corrected for age (Supplementary Tables S3 and S4). The analysis of the entire glycome included 39 N-glycans in plasma (Table 1, Supplementary Figure S1) and 24 IgG N-glycans (Table 2, Supplementary Figure S2).

### 6.1. N-glycans in Plasma

**Figure 1:**
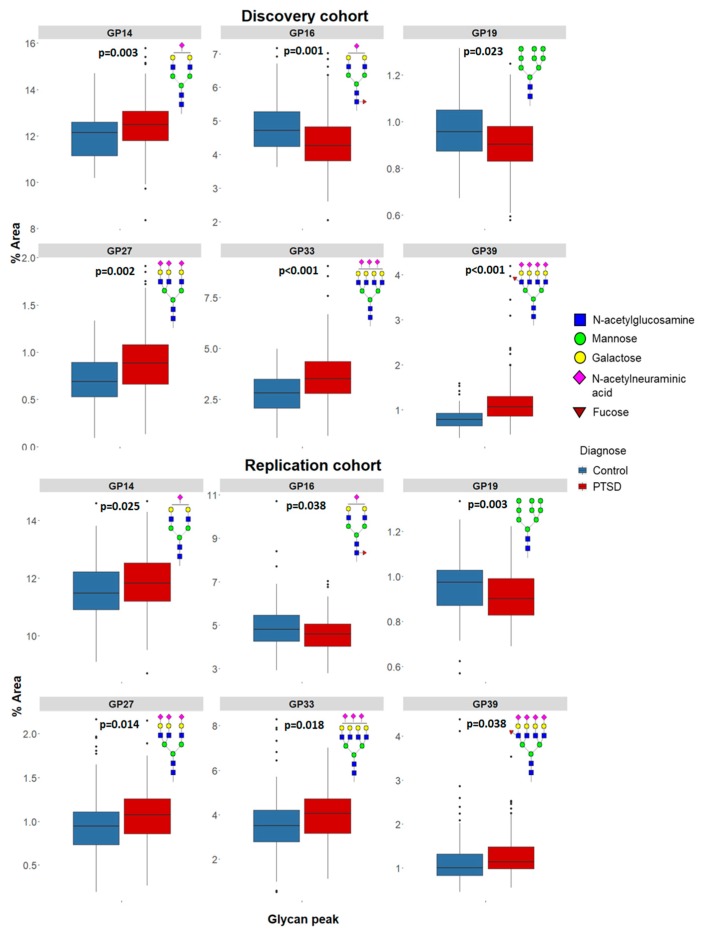
Distribution of significant plasma N-glycans in healthy control subjects and patients with PTSD, in discovery and replication cohort. GP = glycan peak; GP14 = monosialylated glycan (A2G2S1); GP16=monosialylated glycan (FA2G2S1); GP19 = high mannose (M9); GP27 = trisialylated glycan (A3G3S3); GP33 = trisialylated glycan (A4G4S3); GP39 = tetrasialylated glycan (A4F1G4S4); PTSD = post-traumatic stress disorder.

Major glycan peak structures are described in Supplementary Tables S1 and S2. To confirm changes in N-glycan levels in cases/controls, only significantly different N-glycan levels identified in both the discovery and replication cohorts, and altered in the same direction, were designated as significant. In the discovery cohort, comprised of 160 PTSD and 73 age-matched control subjects, 19 out of 39 plasma N-glycans were significantly different between subjects with PTSD and control subjects. In the replication cohort, comprised of 139 PTSD and 171 age-matched control subjects, only six plasma N-glycans out of 19 from the discovery study were confirmed to differ significantly between cases/controls (Table 1). Among these six plasma N-glycans, four (GP14 = A2G2S1, GP27 = A3G3S3, GP33 = A4G4S3, GP39 = A4F1G4S4) were significantly higher, whereas two (GP16 = FA2G2S1, GP19 = M9) were significantly lower in PTSD subjects compared to controls in both cohorts (Figure 1). These results suggested that these six plasma N-glycans might be used as biomarkers of PTSD.

### 6.2. N-glycans Attached to IgG

Only 2 N-glycans bound to IgG (GP6 = FA2B, GP23 = FA2G2S2) differed significantly between individuals with PTSD and healthy subjects in the discovery cohort, but these findings were not confirmed in the replication study (Table 2). These results revealed that N-glycans bound to IgG are not associated with PTSD.

### 6.3. GlycoAge Test

To evaluate whether N-glycans can be used as indicators of the accelerated aging in subjects with PTSD [19], a GlycoAge test was calculated for cases/controls in the discovery/replication study (Table 1; Table 2). There were no significant differences between individuals with PTSD and control subjects in the GlycoAge values in the discovery study for plasma (*p* = 0.208) and IgG N-glycans (*p* = 0.067). These findings were confirmed in the replication study as both plasma (*p* = 0.489) and IgG (*p* = 0.778) GlycoAge values did not differ significantly between participants with PTSD or control participants.

### 6.4. N-glycans and PTSD Severity

As plasma N-glycans differed significantly between cases and controls, we assessed the possible association between plasma N-glycans and PTSD severity using the Spearman’s correlation (Table 3).

In our sample, most of the patients with PTSD had moderate symptoms (92% in both cohorts), whereas the remainder were diagnosed with severe PTSD. No significant correlations were detected between any of the plasma N-glycans and severity of PTSD in discovery or replication cohort (Table 3). Additionally, differences in plasma N-glycans between patients with moderate and severe PTSD, evaluated with Mann–Whitney U test, were not significant. These results confirmed the lack of association between plasma glycosylation and severity of PTSD symptoms (Table 3). 

Furthermore, individuals with moderate and severe PTSD had similar GlycoAge values in the discovery (*p* = 0.984) and replication cohort (*p* = 0.969) (Table 3). These results revealed that PTSD severity was not associated with GlycoAge index in individuals with PTSD. 

## 7. Discussion

### 7.1. N-glycans in Plasma

This is the first study to show significant associations of the plasma, but not IgG, N-glycans with combat-related PTSD. These results, confirmed in both discovery and replication study, have shown in a fairly large sample size, that four plasma N-glycans (A2G2S1, A3G3S3, A4G4S4, A4F1G4S4) were increased, whereas two plasma glycan species (FA2G2S1, M9) were decreased in individuals with PTSD compared to control subjects. Furthermore, individuals with PTSD did not show signs of accelerated physiological aging compared to healthy control subjects, determined with GlycoAge Test index [14,19]. Additionally, severity of PTSD was not associated with plasma N-glycans. 

Although several studies have analyzed N-glycosylation profiles in psychiatric and neurodegenerative diseases [31]; Moreno-Villanueva’s study [19] was the only one that analyzed these changes in PTSD subjects. Their study [19] focused on nine N-glycan structures in plasma of 32 individuals: 13 medicated subjects with PTSD, comorbid with MDD, nine trauma-exposed individuals and 10 low-stress control subjects of different ethnicities. In contrast to our results, they found no significant differences in plasma N-glycans between these groups [19]. However, our study analyzed N-glycosylation profile (39 N-glycan species in plasma and 24 attached to IgG) on a much larger and homogenous sample (*N* = 543) of medication-free males (PTSD/controls), without MDD or other comorbidities.

As N-glycosylation is associated with accelerated aging, a GlycoAge Test has been proposed to predict physiological aging in healthy populations [14]. Higher GlycoAge values have been reported in individuals with AD [14] and PTSD [19], when compared to healthy controls matched for age. Although there was a significant association of GlycoAge values with chronological age in both PTSD and control subjects in our study, there were no significant differences in its values between PTSD/controls, after correcting for age and multiple testing in the large sample of male Caucasian veterans with PTSD. Further modeling of GlycoAge test formula with other identified N-glycan species, obtained in our study, due to more precise separation, and possibly inclusion of additional biochemical and metabolic parameters [32], could yield more accurate results.

Plasma N-glycan profile in PTSD patients from both cohorts was characterized by a greater abundance of highly branched (tri-antennary and tetra-antennary) structures, that were more galactosylated and sialylated (A3G3S3, A4G4S4, and A4F1G4S4). This pattern is identified in acute and chronic inflammation states [33], and in patients 24–48 h after abdominal surgery [34]. Increased highly sialyated and glycosylated N-glycan species (A3F1G3S3 and A4G4LacS4 in low abundant proteins, and A3F1G3S3, A4G4S2, A4G4S3, FA3G3S3, and FA3BG3S3 in highly abundant proteins) were detected in sera of male schizophrenic subjects [15]. Patients with PTSD often exhibit psychotic symptoms [35] that are hallmarks of schizophrenia. Therefore, a similar N-glycosylation profile in these two disorders is not unexpected. A3F1G3S3 N-glycan containing a sLe^x^ epitope, that is associated with increased immunological reaction to antigens [36] and metastasis [37], was significantly increased in both low and high abundant proteins [15]. In our study, A4F1G4S4 N-glycan, that also contains sLe^x^ epitope, was significantly increased in both cohorts, similarly to results in the recently operated patients [34]. Increased glycan branching in total plasma proteins, detected in our veterans with PTSD has previously been found to positively correlate with severity of MDD [16], a common comorbidity in patients with PTSD. Although our veterans did not have clinical MDD, they share some common depressive/numbing symptoms and similar N-glycan changes. In contrast to the results of present study, increased peripheral fucosylation of biantennary glycans and decreased branching, including tri- and tetraantennary glycans, was detected in ADHD in our previous study [17]. These differences might be explained with different diagnoses (ADHD vs. PTSD), differences in gender and age (average 9-year-old male and female children with ADHD vs. 54-year-old male veterans with PTSD and control male participants). 

In our study, high-mannose (M9) and core-fucosylated (FA2G2S1) plasma glycan species were significantly decreased in the PTSD group, compared to the low-stress healthy control, and this result corresponds to lower glycan species found in inflammation [34]. As alternations of high-mannose glycans have been associated with occurrence and progression of different types of cancers [38,39,40], neuropsychiatric disorders [41], inflammation [34] and brain aging [42], whereas PTSD is associated with inflammation [17] and cardiometabolic disorders [4,5], the lower FA2G2S1 glycan result suggests that veterans with PTSD should be closely monitored for these disorders.

Traumatic load was reported to be positively correlated with the GlycoAge Test in subjects with substantial trauma exposure [19]. In our study, severity of PTSD was not significantly associated with plasma N-glycans. This could be explained by the fact that our study included PTSD patients with similar trauma experience and duration, and the fact that 92% of our patients had moderate symptoms of PTSD in both cohorts. 

### 7.2. N-glycans Attached to IgG

We found two IgG glycoforms to be significantly different between PTSD and control subjects in the first cohort. Core fucosylated bisecting agalactosylated N-glycan FA2B was significantly increased in patients with PTSD, while more sialyated and galactosylated form, FA2G2S2, was significantly lower. Core fucosylated N-glycans on IgG are generally considered anti-inflammatory as core fucosylation of IgG can drastically increase antibody-dependent cytotoxicity [43], while decreased galactosylation containing bisecting GlcNAC on IgG is usually hallmark of inflammation and older age [32]. However, the replication cohort did not confirm these changes in FA2B and FA2G2S2 after correcting for multiple testing. Previous research in IgG N-glycome of patients with MDD (male and female combined) has found only two significant glycoforms, both containing bisecting GlcNAC, but similar to our results, there were no significant correlations when males were analyzed separately [16]. 

## 8. Conclusions

We selected two large and balanced cohorts in order to identify potential alterations in N-glycosylation pattern characteristic for PTSD. Strength of the study is in a relatively homogeneous sample of age- and sex-matched PTSD patients and controls, with strict exclusion criteria. To ensure that our findings are due to a true biological variation, and to eliminate any possible experimental artefacts and false positive findings due to chance, we replicated our results using a second cohort. Besides, the study analyzed both plasma and IgG N-glycosylation with high-resolution separation techniques and reported only the results that were significant in both discovery and replication cohort, after adjustment for age and multiple testing. To avoid possible bias, plasma samples in both cohorts were randomized and blinded prior to N-glycan determination. However, we still detected differences between discovery and replication cohort that we cannot yet explain, possibly due to other biological parameters or unknown confounders that we did not take into account during sampling and/or statistical analysis. 

Limitation of this study is in a relatively narrow subpopulation of patients of the same ethnicity, gender and type of trauma. To validate obtained results, inclusion of women, different ethnicities, individuals exposed to different types of trauma, as well as trauma-exposed controls without PTSD is necessary.

In conclusion, this is the first study detecting alterations in the selected plasma N-glycans in PTSD. Our results suggest that PTSD share similar biological underpinning as other neuropsychiatric disorders and inflammation and could point out to some common glycosylation mechanisms underlying neuropsychiatric disorders and inflammation.

## Figures and Tables

**Table 1:** Distribution of total plasma N-glycans in healthy controls and patients with PTSD in discovery and replication cohort. Results are presented as age adjusted percentage of total glycan peak area (median, 25th, and 75th percentile).

Plasma N-glycan Peak	Discovery Cohort (*N* = 233)								Replication Cohort (*N* = 330)							
	Control (*N* = 73)			PTSD (*N* = 160)			Statistics		Control (*N* = 171)			PTSD (*N* = 139)			Statistics	
	Median	25th	75th	Median	25th	75th	MW U	*p*	Median	25th	75th	Median	25th	75th	MW U	*p*
GP1	4.615	3.885	5.514	4.161	3.392	5.103	4810.0	0.062	5.457	4.454	6.559	5.895	4.568	6.843	11094.0	0.524
GP2	1.994	1.740	2.267	1.970	1.726	2.261	5630.0	0.777	2.168	1.911	2.449	2.237	1.901	2.510	11476.0	0.719
GP3	0.084	0.071	0.101	0.089	0.068	0.109	5448.0	0.515	0.103	0.083	0.125	0.106	0.087	0.132	11490.0	0.719
GP4	3.759	3.232	4.411	3.234	2.867	3.711	3783.0	**<0.001**	3.770	3.178	4.426	3.666	3.183	4.308	11793.0	0.908
GP5	2.062	1.658	2.394	1.684	1.394	1.971	3475.0	**<0.001**	2.085	1.645	2.570	1.961	1.617	2.393	10989.0	0.484
GP6	1.212	1.048	1.387	1.157	1.025	1.341	5085.0	0.182	1.336	1.162	1.503	1.336	1.131	1.506	11633.0	0.810
GP7	1.023	0.960	1.124	0.979	0.902	1.144	5235.0	0.304	1.059	0.945	1.180	0.991	0.885	1.110	9285.0	**0.014**
GP8	1.046	0.993	1.134	1.088	0.974	1.189	5260.0	0.310	1.047	0.964	1.173	1.113	1.017	1.246	9453.0	**0.016**
GP9	0.098	0.087	0.112	0.099	0.083	0.113	5827.0	0.986	0.111	0.095	0.127	0.109	0.092	0.126	11156.0	0.545
GP10	3.345	2.983	4.145	2.809	2.313	3.501	3820.0	**<0.001**	3.157	2.529	3.822	3.064	2.511	3.573	11242.0	0.612
GP11	0.701	0.641	0.880	0.701	0.590	0.840	5516.0	0.603	0.820	0.710	0.939	0.757	0.654	0.894	9913.0	**0.043**
GP12	0.998	0.939	1.062	1.022	0.905	1.105	5414.0	0.481	1.005	0.927	1.089	0.972	0.885	1.070	10629.0	0.278
GP13	0.710	0.627	0.875	0.655	0.559	0.761	4210.0	**0.002**	0.801	0.683	0.943	0.775	0.626	0.933	11038.0	0.489
GP14	12.136	11.145	12.590	12.476	11.790	13.067	4261.0	**0.003**	11.478	10.904	12.237	11.823	11.177	12.529	9652.0	**0.025**
GP15	0.396	0.349	0.451	0.443	0.377	0.509	4278.0	**0.003**	0.518	0.448	0.582	0.474	0.427	0.546	9919.0	**0.043**
GP16	4.716	4.240	5.276	4.262	3.813	4.843	4080.0	**0.001**	4.807	4.236	5.467	4.594	4.028	5.060	9813.0	**0.038**
GP17	1.652	1.480	1.977	1.779	1.410	2.272	5254.0	0.310	2.063	1.763	2.404	1.844	1.598	2.224	9375.0	**0.014**
GP18	3.714	3.174	4.006	3.363	3.073	3.765	4873.0	0.082	3.158	2.865	3.495	3.144	2.900	3.619	11353.0	0.712
GP19	0.957	0.873	1.051	0.902	0.829	0.982	4627.0	**0.023**	0.974	0.869	1.030	0.902	0.828	0.991	8778.0	**0.003**
GP20	29.128	27.579	30.740	29.152	27.525	30.769	5657.0	0.802	26.692	24.870	27.838	26.487	24.682	28.160	11505.0	0.719
GP21	0.510	0.466	0.544	0.513	0.468	0.545	5391.0	0.463	0.616	0.560	0.664	0.612	0.557	0.662	11575.0	0.771
GP22	4.243	3.813	4.699	3.859	3.422	4.258	4089.0	**0.001**	4.001	3.607	4.529	3.936	3.459	4.572	10881.0	0.403
GP23	2.009	1.585	2.420	2.029	1.545	2.372	5687.0	0.833	2.281	1.930	2.751	2.163	1.805	2.632	10772.0	0.360
GP24	1.383	1.240	1.725	1.532	1.232	1.814	5061.0	0.171	1.432	1.130	1.693	1.442	1.191	1.739	11457.0	0.719
GP25	0.292	0.269	0.316	0.294	0.270	0.319	5717.0	0.839	0.274	0.252	0.299	0.279	0.247	0.305	11409.0	0.719
GP26	1.434	1.340	1.731	1.621	1.402	1.836	4590.0	**0.020**	1.534	1.322	1.720	1.499	1.275	1.683	10634.0	0.278
GP27	0.690	0.528	0.891	0.884	0.662	1.084	4160.0	**0.002**	0.946	0.734	1.117	1.075	0.853	1.263	9374.0	**0.014**
GP28	0.683	0.589	0.767	0.672	0.551	0.795	5831.0	0.986	0.636	0.520	0.736	0.604	0.508	0.739	11157.0	0.545
GP29	0.201	0.170	0.231	0.184	0.158	0.220	4975.0	0.127	0.181	0.154	0.201	0.172	0.155	0.203	11454.0	0.719
GP30	4.684	4.279	5.584	5.210	4.170	5.869	5029.0	0.156	4.826	3.944	5.490	4.602	3.892	5.347	10852.0	0.397
GP31	0.425	0.358	0.523	0.430	0.339	0.525	5703.0	0.838	0.433	0.350	0.531	0.395	0.320	0.478	9931.0	**0.043**
GP32	1.503	1.199	1.730	1.774	1.468	2.045	3733.0	**<0.001**	1.598	1.344	1.846	1.594	1.315	1.894	11696.0	0.848
GP33	2.807	2.083	3.490	3.523	2.781	4.361	3883.0	**<0.001**	3.504	2.766	4.213	4.049	3.165	4.728	9528.0	**0.018**
GP34	0.347	0.310	0.409	0.395	0.342	0.445	4210.0	**0.002**	0.385	0.340	0.454	0.384	0.347	0.434	11482.0	0.719
GP35	0.352	0.261	0.460	0.424	0.335	0.546	4287.0	**0.003**	0.417	0.346	0.518	0.454	0.369	0.566	10481.0	0.211
GP36	0.488	0.429	0.549	0.603	0.547	0.690	2393.0	**<0.001**	0.557	0.485	0.627	0.593	0.515	0.651	10436.0	0.200
GP37	0.412	0.349	0.473	0.465	0.371	0.568	4533.0	**0.015**	0.452	0.385	0.553	0.462	0.389	0.546	11712.0	0.848
GP38	0.890	0.737	1.013	1.067	0.936	1.258	3029.0	**<0.001**	1.038	0.923	1.222	1.098	0.985	1.234	10786.0	0.360
GP39	0.783	0.645	0.920	1.062	0.856	1.300	2931.0	**<0.001**	1.000	0.826	1.318	1.133	0.974	1.481	9822.0	**0.038**
GlycoAge	0.112	0.017	0.238	0.163	0.040	0.295	5126.0	0.208	0.219	0.128	0.384	0.256	0.145	0.394	11021.0	0.489

Results of the statistics are denoted as Mann Whitney U value (MW U) and false discovery rate (FDR) adjusted *p* value (*p*), where significant results are shown in bold. GP = glycan peak; GlycoAge index = log 10(FA2/FA2G2); PTSD = post-traumatic stress disorder.

**Table 2:** Distribution of N-glycan structures on IgG in healthy controls and patients with PTSD in discovery and replication cohort. Results are presented as age adjusted percentage of total glycan peak area (median, 25th, and 75th percentile).

IgG N-glycan Peak	Discovery cohort (*N* = 233)								Replication cohort (*N* = 330)							
	Control (*N* = 73)			PTSD (*N* = 160)			Statistics		Control (*N* = 171)			PTSD (*N* = 139)			Statistics	
	Median	25th	75th	Median	25th	75th	MW U	*p*	Median	25th	75th	Median	25th	75th	MW U	*p*
GP1	0.085	0.070	0.111	0.089	0.072	0.110	5668.0	0.889	0.097	0.080	0.114	0.100	0.081	0.124	11164.0	0.778
GP2	0.536	0.368	0.719	0.642	0.399	0.924	4887.0	0.170	0.739	0.515	1.001	0.710	0.493	1.111	11671.0	0.987
GP3	0.104	0.088	0.116	0.108	0.091	0.125	5009.0	0.288	0.123	0.111	0.141	0.125	0.110	0.140	11811.0	0.987
GP4	21.850	17.974	25.144	22.733	19.818	27.247	4905.0	0.218	24.042	21.203	28.427	25.366	21.501	29.433	10892.0	0.778
GP5	0.163	0.150	0.178	0.157	0.142	0.174	5214.0	0.218	0.184	0.157	0.209	0.183	0.153	0.214	11700.0	0.894
GP6	5.340	4.262	6.201	6.167	5.056	7.334	4046.0	**0.012**	6.036	4.952	7.274	6.253	5.229	7.436	11067.0	0.778
GP7	0.365	0.289	0.450	0.402	0.274	0.531	5495.0	0.551	0.452	0.333	0.562	0.442	0.354	0.597	11456.0	0.833
GP8	18.936	17.695	19.801	18.551	17.409	19.584	5297.0	0.428	18.179	16.802	19.415	18.129	17.002	19.232	11641.0	0.833
GP9	9.594	8.789	10.605	9.142	8.198	10.011	4672.0	0.061	9.593	8.564	10.422	9.241	8.488	10.050	10592.0	0.778
GP10	4.910	4.291	5.618	5.172	4.574	6.129	4688.0	0.067	5.083	4.405	5.728	5.134	4.342	6.037	11427.0	0.833
GP11	0.670	0.578	0.732	0.708	0.638	0.821	4489.0	0.061	0.683	0.612	0.756	0.678	0.593	0.765	11853.0	0.987
GP12	0.684	0.544	0.895	0.643	0.466	0.928	5521.0	0.730	0.607	0.456	0.923	0.615	0.477	0.966	11571.0	0.833
GP13	0.264	0.230	0.298	0.258	0.229	0.293	5688.0	0.885	0.261	0.228	0.297	0.257	0.225	0.300	11484.0	0.833
GP14	13.171	11.025	15.405	11.864	9.800	13.913	4284.0	0.051	11.689	8.929	13.593	11.015	8.708	12.681	11005.0	0.778
GP15	1.762	1.595	1.919	1.736	1.482	1.960	5570.0	0.885	1.651	1.445	1.861	1.639	1.438	1.849	11831.0	0.987
GP16	3.126	2.837	3.437	3.086	2.768	3.393	5539.0	0.430	3.086	2.838	3.429	3.071	2.739	3.406	11222.0	0.778
GP17	0.973	0.917	1.088	0.967	0.881	1.110	5638.0	0.885	1.008	0.878	1.125	0.986	0.882	1.119	11677.0	0.833
GP18	9.359	7.857	10.641	8.559	6.916	9.896	4443.0	0.061	7.824	6.701	9.282	7.705	6.738	8.886	11115.0	0.778
GP19	1.992	1.749	2.206	2.010	1.752	2.314	5461.0	0.612	1.966	1.762	2.259	1.952	1.602	2.207	10479.0	0.373
GP20	0.419	0.373	0.471	0.428	0.380	0.472	5837.0	0.885	0.404	0.362	0.460	0.398	0.353	0.455	11154.0	0.778
GP21	0.851	0.783	0.942	0.875	0.792	0.972	5459.0	0.430	0.908	0.834	1.003	0.874	0.802	0.958	9792.0	0.174
GP22	0.165	0.132	0.201	0.173	0.143	0.210	5273.0	0.327	0.148	0.122	0.182	0.146	0.120	0.178	11278.0	0.778
GP23	1.790	1.508	2.226	1.587	1.340	1.888	4307.0	**0.025**	1.615	1.426	2.035	1.623	1.303	1.847	10358.0	0.373
GP24	1.816	1.637	2.070	1.939	1.669	2.253	5011.0	0.259	1.960	1.735	2.229	1.865	1.559	2.241	10417.0	0.373
Glycoage	0.211	0.053	0.335	0.283	0.155	0.446	4735.0	0.067	0.313	0.207	0.521	0.354	0.245	0.520	11271.0	0.778

Results of the statistics are denoted as Mann Whitney U value (MW U) and adjusted *p*-value (false discovery rate (FDR) method). GP = glycan peak; GlycoAge index = log 10(FA2/FA2G2); PTSD: post-traumatic stress disorder.

**Table 3:** Correlation between PTSD severity determined by the CAPS scores and distribution of total plasma N-glycan structures, as well as differences between veterans with moderate and severe PTSD in discovery and replication cohort.

Plasma N-Glycan Peak	Discovery Cohort (*N* = 233)				Replication Cohort (*N* = 330)			
	rho	*p*	MW U	*p*	rho	*p*	MW U	*p*
GP1	0.035	0.791	875.0	0.984	0.076	0.991	612.0	0.921
GP2	0.031	0.801	885.0	0.984	0.009	0.991	644.0	0.921
GP3	0.126	0.364	804.0	0.899	0.078	0.991	562.0	0.921
GP4	0.139	0.330	821.0	0.899	0.003	0.991	615.0	0.921
GP5	0.149	0.330	815.0	0.899	0.034	0.991	576.0	0.921
GP6	0.104	0.388	741.0	0.829	0.076	0.991	683.0	0.969
GP7	0.095	0.395	753.0	0.829	0.119	0.991	687.0	0.969
GP8	0.026	0.826	693.0	0.829	0.009	0.991	649.0	0.921
GP9	0.111	0.388	816.0	0.899	0.037	0.991	668.0	0.969
GP10	0.110	0.388	877.0	0.984	0.017	0.991	635.0	0.921
GP11	0.055	0.735	686.0	0.829	0.060	0.991	699.0	0.969
GP12	0.013	0.904	680.0	0.829	0.020	0.991	648.0	0.921
GP13	0.110	0.388	839.0	0.969	0.020	0.991	508.0	0.921
GP14	0.047	0.773	678.0	0.829	0.005	0.991	570.0	0.921
GP15	0.100	0.388	817.0	0.899	0.014	0.991	525.0	0.921
GP16	0.100	0.388	770.0	0.829	0.012	0.991	544.0	0.921
GP17	0.044	0.784	735.0	0.829	0.018	0.991	641.0	0.921
GP18	0.041	0.791	745.0	0.829	0.001	0.991	685.0	0.969
GP19	0.148	0.330	602.0	0.829	0.204	0.312	593.0	0.921
GP20	0.118	0.388	798.0	0.899	0.004	0.991	605.0	0.921
GP21	0.034	0.791	881.0	0.984	0.092	0.991	625.0	0.921
GP22	0.095	0.395	845.0	0.976	0.029	0.991	605.0	0.921
GP23	0.012	0.904	850.0	0.977	0.097	0.991	514.0	0.921
GP24	0.137	0.330	823.0	0.899	0.025	0.991	696.0	0.969
GP25	0.034	0.791	635.0	0.829	0.205	0.312	505.0	0.921
GP26	0.048	0.773	685.0	0.829	0.070	0.991	586.0	0.921
GP27	0.136	0.330	725.0	0.829	0.064	0.991	452.0	0.921
GP28	0.170	0.330	736.0	0.829	0.012	0.991	676.0	0.969
GP29	0.009	0.914	770.0	0.829	0.058	0.991	672.0	0.969
GP30	0.133	0.330	621.0	0.829	0.018	0.991	693.0	0.969
GP31	0.158	0.330	668.0	0.829	0.117	0.991	641.0	0.921
GP32	0.104	0.388	872.0	0.984	0.008	0.991	602.0	0.921
GP33	0.146	0.330	760.0	0.829	0.011	0.991	508.0	0.921
GP34	0.017	0.901	752.0	0.829	0.134	0.991	590.0	0.921
GP35	0.106	0.388	773.0	0.829	0.038	0.991	515.0	0.921
GP36	0.136	0.330	867.0	0.984	0.093	0.991	580.0	0.921
GP37	0.056	0.735	696.0	0.829	0.002	0.991	614.0	0.921
GP38	0.074	0.575	772.0	0.829	0.028	0.991	695.0	0.969
GP39	0.151	0.330	806.0	0.899	0.025	0.991	628.0	0.921
GlycoAge	0.036	0.791	875.0	0.984	0.013	0.991	689.0	0.969

Results of the statistics are denoted as Spearman’s rho coefficient (ρ), Mann Whitney U value (MW U), and corresponding adjusted *p* value (false discovery rate (FDR) method). GP = glycan peak; GlycoAge index = log10 (FA2/FA2G2); PTSD = post-traumatic stress disorder.

## Supplementary Materials

The following are available online at https://www.mdpi.com/2218-273X/9/12/834/s1, Figure S1: Representative chromatogram of plasma N-glycans, Figure S2: Representative chromatogram of IgG N-glycans, Table S1: Plasma N-glycan peaks separated by HILIC-UPLC and their composition as described by Gudelj et al. (2016), Table S2: IgG N-glycan peaks separated by HILIC-UPLC and their composition as described by Nikolac Perkovic et al. (2016), Table S3: Effects of age, smoking and BMI on plasma N-glycan species in the discovery and replication cohort, Table S4. Effects of age, smoking and BMI on plasma N-glycan species in the discovery and replication cohort.
