# Supplementary material for: N-glycomic Profile in Combat Related Post-Traumatic Stress Disorder
Source: Biomolecules. 2019 Dec 6;9(12):834. doi: 10.3390/biom9120834 (PMC6995522; doi:10.3390/biom9120834)

Supplementary Table S1. Plasma N-glycan peaks separated by HILIC-UPLC and their composition as described by Gudelj et al. (2016)

Glycan peak	Major glycan structure	Description
GP1	FA2	agalactosylated
GP2	M5, FA2B	high mannose (M5), agalactosylated with bisecting GlcNAc (FA2B)
GP3	A2[6]BG1	monogalactosylated with bisecting GlcNAc
GP4	FA2[6]G1	monogalactosylated with core fucose
GP5	FA2[3]G1	monogalactosylated with core fucose
GP6	FA2[6]BG1	monogalactosylated with core fucose and bisecting GlcNAc
GP7	M6	high mannose
GP8	A2G2	Digalactosylated
GP9	A2BG2	digalactosylated with bisecting GlcNAc
GP10	FA2G2	digalactosylated with core fucose
GP11	FA2BG2	digalactosylated with core fucose and bisecting GlcNAc
GP12	A2[3]BG1S1	monogalactosylated with bisecting GlcNAc
GP13	FA2[3]G1S1	monogalactosylated and sialylated with core fucose
GP14	A2G2S1	digalactosylated and sialylated
GP15	A2BG2S1	digalactosylated and sialylated with bisecting GlcNAc
GP16	FA2G2S1	digalactosylated and sialylated with core fucose
GP17	FA2BG2S1	digalactosylated and sialylated with bisecting GlcNAc and core fucose
GP18	A2G2S2	digalactosylated and disialylated
GP19	M9	high mannose
GP20	A2G2S2	digalactosylated and disialylated
GP21	A2BG2S2	digalactosylated and disialylated with bisecting GlcNAc
GP22	FA2G2S2	digalactosylated and disialylated with core fucose
GP23	FA2BG2S2	digalactosylated and disialylated with bisecting GlcNAc and core fucose
GP24	A3G3S2	trigalactosylated and disialylated
GP25	A3BG3S2	trigalactosylated and disialylated with bisecting GlcNAc
GP26	A3G3S2	trigalactosylated and disialylated
GP27	A3G3S3	trigalactosylated and trisialylated
GP28	A3G3S3	trigalactosylated and trisialylated
GP29	FA3G3S3	trigalactosylated and trisialylated with core fucose
GP30	A3G3S3	trigalactosylated and trisialylated
GP31	FA3G3S3	trigalactosylated and trisialylated with core fucose
GP32	A3F1G3S3	trigalactosylated and trisialylated with antennary fucose
GP33	A4G4S3	tetragalactosylated and trisialylated
GP34	A4G4S3	tetragalactosylated and trisialylated
GP35	A4F1G3S3	tetragalactosylated and trisialylated with antennary fucose
GP36	A4G4S4	tetragalactosylated and tetrasialylated
GP37	A4G4S4	tetragalactosylated and tetrasialylated
GP38	A4G4S4	tetragalactosylated and tetrasialylated
GP39	A4F1G4S4	tetragalactosylated and tetrasialylated with antennary fucose

Supplementary Table S2. IgG N-glycan peaks separated by HILIC-UPLC and their composition as described by Nikolac Perkovic et al. (2016)

Glycan peak	Major glycan structure	Description
GP1	FA1	agalactosylated with core fucose
GP2	A2	agalactosylated
GP3	A2B	agalactosylated with bisecting GlcNAc
GP4	FA2	agalactosylated with core fucose
GP5	M5	high mannose
GP6	FA2B	agalactosylated with core fucose and bisecting GlcNAc
GP7	A2G1	monogalactosylated
GP8	FA2[6]G1	monogalactosylated with core fucose
GP9	FA2[3]G1	monogalactosylated with core fucose
GP10	FA2[6]BG1	monogalactosylated with core fucose and bisecting GlcNAc
GP11	FA2[3]BG1	monogalactosylated with core fucose and bisecting GlcNAc
GP12	A2G2	digalactosylated
GP13	A2BG2	digalactosylated with bisecting GlcNAc
GP14	FA2G2	digalactosylated with core fucose
GP15	FA2BG2	digalactosylated with core fucose and bisecting GlcNAc
GP16	FA2G1S1	monogalactosylated and sialyated with core fucose
GP17	A2G2S1	digalactosylated and sialyated
GP18	FA2G2S1	digalactosylated and sialyated with core fucose
GP19	FA2BG2S1	digalactosylated and sialyated with core fucose and bisecting GlcNAc
GP20	n.d.	structure not determined
GP21	A2G2S2	digalactosylated and disialyated
GP22	A2BG2S2	digalactosylated and disialyated with bisecting GlcNAc
GP23	FA2G2S2	digalactosylated and disialyated with core fucose
GP24	FA2BG2S2	digalactosylated and disialyated with core fucose and bisecting GlcNAc

Supplementary Table S3. Effects of age, smoking and BMI on plasma N-glycan species in the discovery and replication cohort. Significance of the regression models are represented as <0.05, 0.001 or non-significant (NS).

Plasma glycan peak	Discovery cohort								Replication cohort							
	Age		Smoking		BMI		Model		Age		Smoking		BMI		Model	
	β	p	β	p	β	p	*R^2	p	β	p	β	p	β	p	*R^2	p
GP1	0.031	0.047	0.241	0.238	-0.055	0.084	0.024	<0.05	0.029	0.025	0.276	0.182	0.028	0.279	0.015	NS
GP2	0.006	0.212	0.045	0.481	-0.013	0.191	0.003	NS	0.009	0.005	-0.042	0.421	0.003	0.625	0.021	<0.05
GP3	0.001	0.228	-0.005	0.272	-0.001	0.121	0.009	NS	0.001	<0.001	-0.001	0.720	0.000	0.806	0.060	<0.001
GP4	-0.012	0.198	0.114	0.365	-0.003	0.876	0.001	NS	-0.014	0.020	0.056	0.571	0.016	0.191	0.015	NS
GP5	-0.008	0.141	0.082	0.237	0.001	0.979	0.004	NS	-0.012	0.002	0.088	0.154	0.013	0.088	0.042	<0.001
GP6	0.001	0.858	0.015	0.711	0.004	0.552	0.002	NS	0.001	0.707	-0.021	0.526	0.009	0.038	0.006	NS
GP7	-0.005	0.106	0.061	0.103	0.003	0.560	0.014	NS	-0.005	0.001	0.000	0.999	0.004	0.141	0.032	<0.001
GP8	-0.002	0.140	-0.004	0.838	-0.004	0.228	0.004	NS	0.003	0.012	-0.038	0.046	-0.003	0.211	0.032	<0.001
GP9	-0.000	0.132	-0.001	0.824	-0.000	0.773	0.011	NS	0.000	0.038	-0.005	0.119	0.000	0.697	0.015	NS
GP10	-0.036	<0.001	0.126	0.341	0.008	0.702	0.052	<0.001	-0.025	<0.001	-0.021	0.830	0.004	0.772	0.047	<0.001
GP11	-0.005	0.045	0.018	0.586	0.004	0.493	0.009	NS	-0.003	0.031	-0.022	0.396	0.004	0.167	0.012	NS
GP12	-0.002	0.327	0.001	0.951	0.001	0.682	0.001	NS	0.002	0.149	-0.024	0.157	0.000	0.927	0.005	NS
GP13	-0.000	0.870	0.039	0.08	-0.004	0.205	0.011	NS	-0.001	0.301	0.043	0.054	0.003	0.219	0.012	NS
GP14	0.001	0.922	-0.026	0.871	-0.011	0.663	0.001	NS	0.020	0.007	-0.100	0.407	-0.022	0.161	0.024	<0.05
GP15	-0.001	0.350	0.006	0.698	0.001	0.735	0.001	NS	0.001	0.390	-0.007	0.593	0.001	0.456	-0.004	NS
GP16	-0.030	0.002	0.209	0.092	0.008	0.672	0.050	<0.001	-0.030	<0.001	0.091	0.375	0.020	0.122	0.074	<0.001
GP17	-0.006	0.479	0.063	0.563	0.008	0.654	0.001	NS	-0.006	0.224	-0.030	0.711	0.008	0.467	-0.003	NS
GP18	-0.012	0.041	-0.094	0.235	0.009	0.439	0.014	NS	-0.003	0.386	-0.062	0.293	-0.032	<0.001	0.052	<0.001
GP19	-0.002	0.155	0.036	0.047	0.001	0.857	0.016	NS	-0.004	<0.001	0.013	0.314	0.001	0.407	0.087	<0.001
GP20	0.009	0.721	-0.266	0.442	0.065	0.223	0.001	NS	-0.019	0.238	-0.035	0.893	-0.005	0.873	-0.005	NS
GP21	-0.001	0.140	-0.007	0.483	0.000	0.802	-0.002	NS	0.000	0.543	0.002	0.820	-0.001	0.408	-0.006	NS
GP22	0.004	0.558	0.128	0.192	0.002	0.880	-0.005	NS	-0.012	0.023	0.073	0.380	0.020	0.059	0.022	<0.05
GP23	0.010	0.190	-0.040	0.702	-0.008	0.626	-0.004	NS	0.009	0.082	0.085	0.288	0.006	0.585	0.004	NS
GP24	-0.003	0.450	-0.009	0.879	0.005	0.586	-0.010	NS	-0.000	0.893	-0.062	0.197	-0.016	0.011	0.017	<0.05
GP25	0.000	0.604	-0.003	0.694	0.000	0.618	-0.011	NS	0.000	0.658	-0.002	0.722	-0.002	0.001	0.029	<0.001
GP26	0.001	0.666	0.025	0.576	0.004	0.536	-0.010	NS	0.001	0.716	-0.023	0.507	0.002	0.705	-0.007	NS
GP27	0.007	0.029	-0.096	0.033	-0.010	0.166	0.038	<0.05	0.008	0.002	-0.005	0.899	-0.002	0.741	0.024	<0.05
GP28	-0.002	0.241	0.006	0.819	0.004	0.286	-0.002	NS	-0.001	0.293	-0.022	0.252	-0.007	0.008	0.021	<0.05
GP29	-0.000	0.511	-0.008	0.183	0.000	0.697	-0.003	NS	-0.000	0.961	-0.008	0.084	-0.002	0.005	0.026	<0.05
GP30	-0.008	0.549	0.038	0.827	0.040	0.133	-0.002	NS	-0.010	0.243	-0.080	0.553	-0.036	0.036	0.010	NS
GP31	-0.000	0.806	0.024	0.260	0.005	0.125	0.002	NS	-0.001	0.423	-0.019	0.189	-0.001	0.733	-0.002	NS

GP32	0.010	0.030	-0.035	0.575	-0.002	0.847	0.011	NS	0.003	0.247	-0.010	0.828	0.011	0.062	0.007	NS
GP33	0.032	0.021	-0.428	0.020	-0.036	0.193	0.045	<0.001	0.027	0.003	-0.004	0.981	-0.007	0.703	0.020	<0.05
GP34	0.001	0.255	-0.001	0.956	0.000	0.811	-0.007	NS	0.001	0.039	-0.013	0.164	0.0002	0.876	0.013	NS
GP35	0.004	0.017	-0.035	0.120	-0.003	0.379	0.029	<0.05	0.004	0.001	-0.011	0.571	0.0002	0.943	0.031	<0.001
GP36	0.002	0.086	-0.038	0.040	-0.000	0.932	0.023	NS	0.003	<0.001	-0.030	0.020	-0.002	0.260	0.073	<0.001
GP37	-0.001	0.356	-0.021	0.326	0.003	0.333	-0.001	NS	0.001	0.322	-0.029	0.087	-0.007	0.002	0.035	<0.001
GP38	0.003	0.196	-0.082	0.022	0.001	0.886	0.022	NS	0.006	0.001	-0.052	0.071	-0.008	0.028	0.055	<0.001
GP39	0.012	0.026	-0.160	0.026	-0.013	0.232	0.040	<0.001	0.016	<0.001	-0.040	0.481	-0.006	0.404	0.057	<0.001
GlycoAge	0.008	<0.001	0.006	0.809	-0.006	0.112	0.065	<0.001	0.006	<0.001	0.027	0.220	0.002	0.563	0.056	<0.001

BMI – body mass index, NS – non significant; Glycoage – log10(FA2/FA2G2); *R^2 – adjusted R^2, β - beta coefficient

Supplementary Table S4. Effects of age, smoking and BMI on plasma N-glycan species in the discovery and replication cohort. Significance of the regression models are represented as <0.05, 0.001 or non-significant (NS).

IgG glycan peak	Discovery cohort								Replication cohort							
	Age		Smoking		BMI		Model		Age		Smoking		BMI		Model	
	β	p	β	p	β	p	*R^2	p	β	p	β	p	β	p	*R^2	p
GP1	0.001	0.051	0.002	0.660	-0.001	0.165	0.013	NS	0.001	0.032	0.003	0.484	-0.001	0.157	0.012	NS
GP2	0.080	0.067	-0.012	0.838	-0.008	0.382	0.005	NS	0.008	0.006	-0.036	0.457	-0.012	0.045	0.031	<0.001
GP3	0.001	0.006	0.000	0.894	-0.000	0.582	0.024	<0.05	0.001	0.003	0.008	0.035	0.000	0.754	0.030	<0.001
GP4	0.210	0.001	0.352	0.664	-0.222	0.075	0.052	<0.001	0.192	<0.001	0.959	0.168	0.013	0.884	0.055	<0.001
GP5	0.001	0.058	0.002	0.664	0.000	0.870	0.004	NS	0.000	0.184	0.003	0.561	-0.001	0.427	-0.001	NS
GP6	0.047	0.009	-0.093	0.696	-0.031	0.397	0.022	NS	0.051	<0.001	-0.228	0.194	-0.016	0.487	0.068	<0.001
GP7	-0.001	0.808	-0.017	0.549	-0.002	0.643	-0.012	NS	0.001	0.919	-0.013	0.545	-0.005	0.087	0.001	NS
GP8	-0.002	0.909	-0.111	0.682	0.038	0.361	-0.009	NS	-0.029	0.023	-0.044	0.832	0.020	0.449	0.009	NS
GP9	-0.001	0.943	0.030	0.869	0.024	0.390	-0.011	NS	-0.034	<0.001	0.190	0.204	0.015	0.420	0.043	<0.001
GP10	0.014	0.224	-0.112	0.477	0.042	0.086	0.012	NS	0.005	0.500	-0.297	0.016	0.017	0.264	0.016	<0.05
GP11	0.003	0.067	-0.017	0.373	0.002	0.448	0.011	NS	0.001	0.145	-0.013	0.366	0.001	0.502	0.003	NS
GP12	-0.012	0.007	-0.051	0.363	0.000	0.993	0.023	<0.05	-0.005	0.121	-0.051	0.285	-0.008	0.196	0.007	NS
GP13	-0.001	0.116	-0.011	0.161	0.001	0.300	0.011	NS	-0.000	0.256	-0.007	0.29	-0.001	0.285	0.001	NS
GP14	-0.157	<0.001	0.100	0.823	0.111	0.107	0.091	<0.001	-0.117	<0.001	-0.297	0.383	-0.018	0.683	0.086	<0.001
GP15	-0.011	0.004	-0.053	0.283	0.016	0.041	0.047	<0.001	-0.007	0.003	-0.076	0.052	-0.000	0.970	0.028	<0.001
GP16	0.006	0.196	0.025	0.679	-0.008	0.383	-0.002	NS	-0.003	0.280	0.046	0.377	0.007	0.294	0.001	NS
GP17	-0.003	0.147	-0.021	0.471	-0.002	0.691	-0.002	NS	0.002	0.190	-0.011	0.724	-0.005	0.236	0.001	NS
GP18	-0.092	<0.001	0.130	0.675	0.029	0.537	0.059	<0.001	-0.069	<0.001	-0.115	0.593	-0.003	0.900	0.074	<0.001
GP19	-0.0004	0.933	-0.091	0.166	0.008	0.385	0.003	NS	0.003	0.297	-0.003	0.950	-0.000	0.965	-0.006	NS
GP20	-0.001	0.448	-0.000	0.988	0.001	0.570	-0.010	NS	-0.001	0.193	0.004	0.737	0.000	0.804	-0.003	NS
GP21	-0.001	0.458	-0.006	0.802	0.008	0.025	0.013	NS	0.000	0.810	-0.029	0.122	0.002	0.363	0.001	NS
GP22	-0.0001	0.865	-0.003	0.626	-0.001	0.611	-0.012	NS	0.001	0.050	-0.011	0.169	-0.001	0.464	0.013	NS
GP23	-0.009	0.067	0.085	0.174	0.002	0.810	0.013	NS	-0.008	0.008	0.051	0.310	-0.001	0.86	0.019	<0.05
GP24	0.005	0.300	-0.068	0.263	-0.002	0.811	-0.002	NS	0.008	0.026	-0.045	0.438	-0.001	0.847	0.010	NS
Glycoage	0.010	<0.001	0.004	0.907	-0.009	0.070	0.078	<0.001	0.008	<0.001	0.031	0.221	0.001	0.881	0.074	<0.001

BMI – body mass index; NS – non significant; Glycoage – log10(FA2/FA2G2); *R^2 – adjusted R^2, β - beta coefficient

Figure S1: Representative chromatogram of plasma N-glycans.

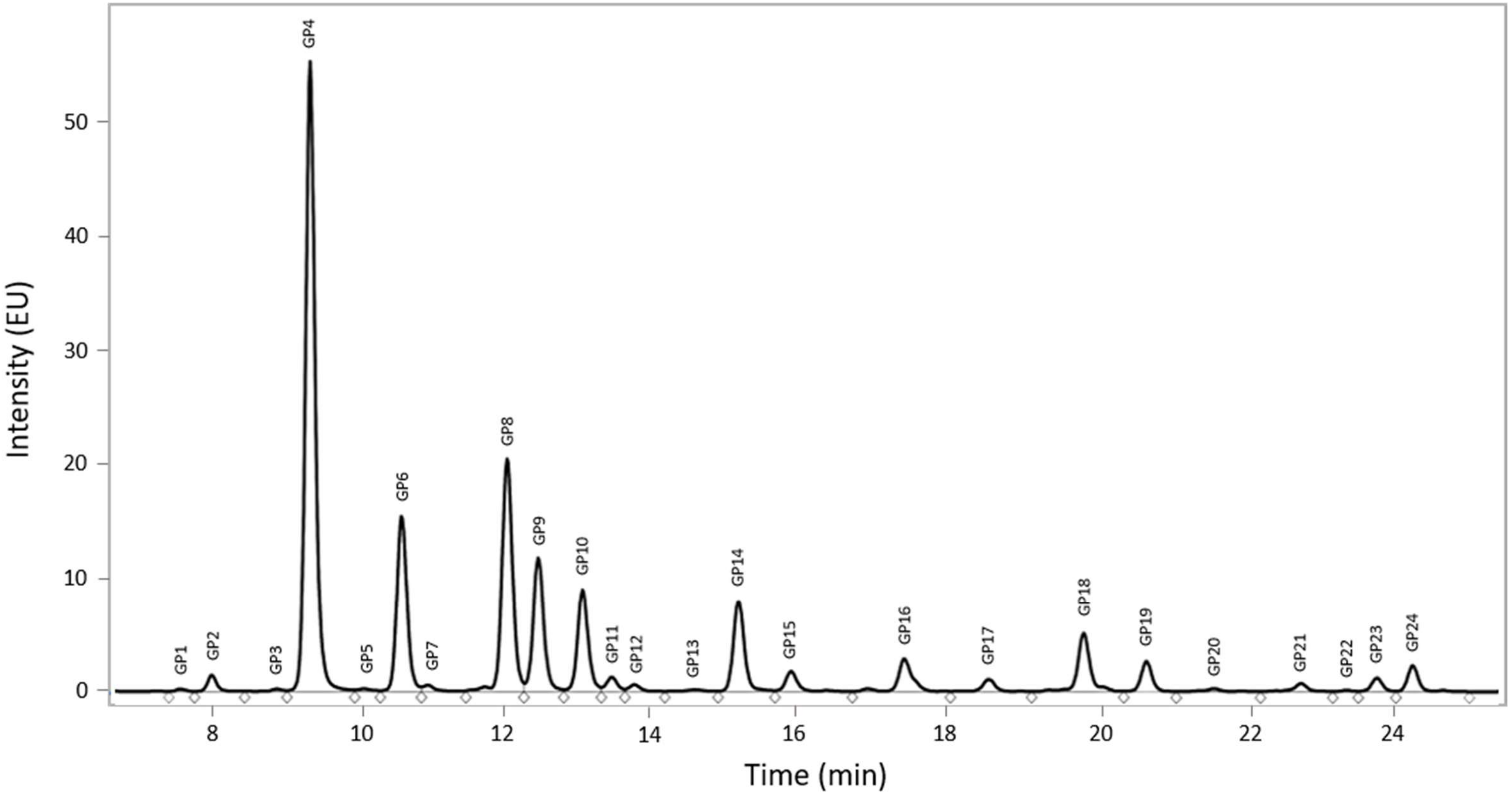

Figure S2: Representative chromatogram of IgG N-glycans.

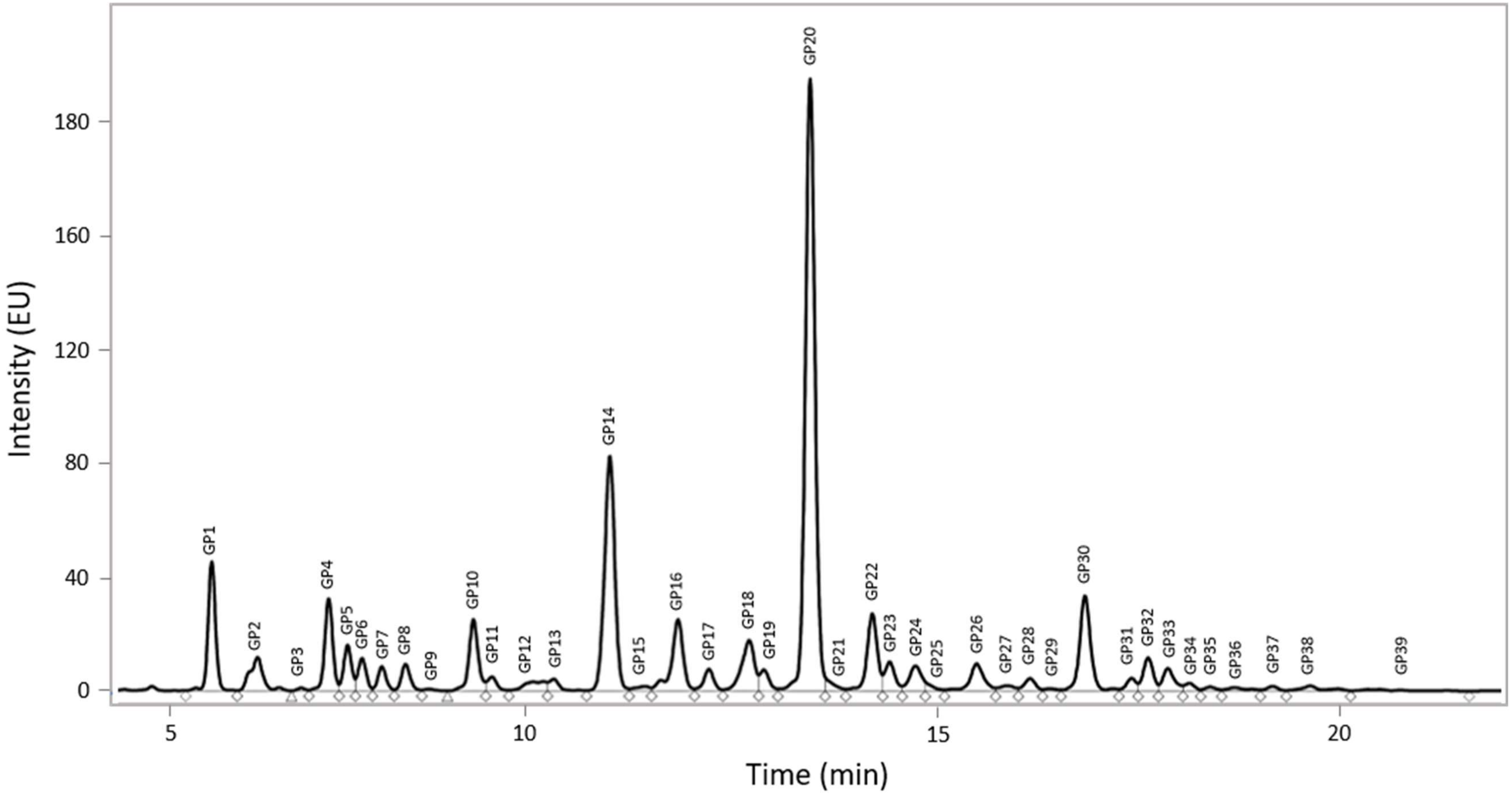

Supplement: Supplementary file 1 [file biomolecules-09-00834-s001.pdf]
